# Association between forgone care and household income among the elderly in five Western European countries – analyses based on survey data from the SHARE-study

**DOI:** 10.1186/1472-6963-9-52

**Published:** 2009-03-23

**Authors:** Andreas Mielck, Raphael Kiess, Olaf von dem Knesebeck, Irina Stirbu, Anton E Kunst

**Affiliations:** 1 Helmholtz Zentrum Muenchen – German Research Center for Environmental Health, Institute of Health Economics and Health Care Management, P.O. Box 1129, 85758 Neuherberg, Germany; 2 University Medical Center Hamburg-Eppendorf, Department of Medical Sociology, Martinistr. 52, 20246 Hamburg, Germany; 3 Erasmus Medical Center, Department of Public Health, P.O. Box 2040, 3000 CA, Rotterdam, The Netherlands

## Abstract

**Background:**

Studies on the association between access to health care and household income have rarely included an assessment of 'forgone care', but this indicator could add to our understanding of the inverse care law. We hypothesize that reporting forgone care is more prevalent in low income groups.

**Methods:**

The study is based on the 'Survey of Health, Ageing and Retirement in Europe (SHARE)', focusing on the non-institutionalized population aged 50 years or older. Data are included from France, Germany, Greece, Italy and Sweden. The dependent variable is assessed by the following question: During the last twelve months, did you forgo any types of care because of the costs you would have to pay, or because this care was not available or not easily accessible? The main independent variable is household income, adjusted for household size and split into quintiles, calculating the quintile limits for each country separately. Information on age, sex, self assessed health and chronic disease is included as well. Logistic regression models were used for the multivariate analyses.

**Results:**

The overall level of forgone care differs considerably between the five countries (e.g. about 10 percent in Greece and 6 percent in Sweden). Low income groups report forgone care more often than high income groups. This association can also be found in analyses restricted to the subsample of persons with chronic disease. Associations between forgone care and income are particularly strong in Germany and Greece. Taking the example of Germany, forgone care in the lowest income quintile is 1.98 times (95% CI: 1.08–3.63) as high as in the highest income quintile.

**Conclusion:**

Forgone care should be reduced even if it is not justified by an 'objective' need for health care, as it could be an independent stressor in its own right, and as patient satisfaction is a strong predictor of compliance. These efforts should focus on population groups with particularly high prevalence of forgone care, for example on patients with poor self assessed health, on women, and on low income groups. The inter-country differences point to the need to specify different policy recommendations for different countries.

## Background

Issues of forgone care could provide an important link between health inequalities and health care provision. Empirical studies in this field have rarely included the explicit statement that some care has been forgone due to financial problems or unavailability. There are only few studies focusing on these issues, and most of them are from the USA [e.g. [1-8]]. They indicate that forgone care is experienced mostly by children and adults from the low socio-economic status groups (e.g. low income, lack of insurance coverage, ethnic and racial minorities). The assessment of 'forgone care' is usually based on self reports, i.e. the respondents are asked if they did not obtain medical care which they believed they had needed.

Even though this is a subjective statement, it clearly indicates an important discomfort with the health care system, and of course it could also indicate a lost chance for improving the health status. These analyses concerning differences between the need for health care and the actual provision could also be important for improving our understanding of health inequalities. At least in Western Europe, health inequalities are mostly explained by individual health behaviour (smoking, physical activity, diet etc.). It has often been stressed, though, that other determinants should be included as well [e.g. [9-13]]. This discussion usually focuses on living and working conditions and on the social environment, not on the health care system. Indicators of health care provision and utilization could represent important entry points for interventions aimed at reducing health inequalities. On one hand, health care should be provided for all who could benefit, thus potentially reducing health inequalities. On the other, health care expenditures have to be limited. Empirical evidence concerning social inequalities in health care is essential for finding the balance between these two objectives. To date this empirical basis is not very strong, though, at least in Western Europe.

Analysing the association between forgone care and household income in more detail could be an important contribution to our understanding of the inverse care law [14,15]. It states "that the availability of good medical care tends to vary inversely with the need for it in the population served" [[14], p. 405]. Poor health care could be expressed in many different ways. One example is poor quality of health care actually delivered, but this can hardly be assessed in surveys including questions on forgone care. Other examples are poor regional access to health care providers, long waiting lists, high prices and co-payments. They could all result in forgone care, with the potential of creating health care inequalities favouring the upper status groups.

## Methods

The study is based on the 'Survey of Health, Ageing and Retirement in Europe (SHARE)', focusing on the non-institutionalized population aged 50 years or older from 11 European countries plus Israel [16-23]. The data analysed here are taken from release 2.0.1 (released July 5, 2007) of the SHARE dataset, including data from 27,519 adults from 11 European countries. Based on probability samples in each participating country, data were collected in 2004 using a computer assisted personal interviewing (CAPI) programme, supplemented by a self-completion paper and pencil questionnaire. The country-specific household response rates vary considerably, with eight countries having rates above 50 percent. Due to the very low response rates for Switzerland (38.8) and Belgium (39.2%), these two countries were excluded from further analyses.

The analyses presented below focus on the dependent variable 'forgone care'. The corresponding questions read: (a) During the last twelve months, did you forgo any types of care because of the costs you would have to pay? (yes/no); (b) During the last twelve months, did you forgo any types of care because they were not available or not easily accessible? (yes/no). Both questions were asked in the computer assisted personal interview. Due to small numbers an aggregated variable (forgone care because of costs 'or' because care is unavailable) is used in most analyses. Another reason for combining these two questions is that it could be difficult for the respondents to clearly differentiate between these two potential reasons for forgone care.

In our analyses the independent variable of primary importance is household income. In the SHARE dataset, the variable 'gross total annual household income for 2003' has been calculated in the following way: sum of the gross individual income of each household member (income from employment, self-employment, pension, private regular transfers such as alimony, long term care), of capital assets income (income from bank accounts, bonds, stocks) and of rent payments received, excluding imputed rent from owner-occupied housing [16].

In order to adjust for household size, we applied a formula that has been used in the Luxembourg income study [24] and that has been proposed for studies on health inequalities [25]: adjusted per capita income = gross annual household income, divided by the number of household members to the power of 0.36 (income/household size*^0.36^). For further analyses, we have split the adjusted per capita income into quintiles, calculating the quintile limits for each country separately.

Information on age, sex, self assessed health and chronic disease is included as well. Concerning age, three groups are distinguished, more or less representing tertiles when all countries are combined (50–58 years, 59–67 years, 68 years or older). The information on self assessed health (SAH) is derived from the following questions: "Would you say your health is..?" (categories: very good, good, fair, bad, very bad). We have combined the first two categories (summarized as 'good') and the last two categories (summarized as 'bad'). We have restricted some analyses to persons with a chronic disease; the prevalence was assessed by the following question: "Has a doctor ever told you that you had any of the following conditions?" Fourteen physician diagnosed diseases were listed, and we excluded only those two that could indicate minor health problems (i.e. high blood pressure or hypertension, high blood cholesterol). Thus, chronic disease was defined as having at least one of the remaining twelve conditions: heart attack (including myocardial infarction or coronary thrombosis, or any other heart problem including congestive heart failure), stroke or cerebral vascular disease, diabetes or high blood sugar, chronic lung disease (such as chronic bronchitis or emphysema), asthma, arthritis (including osteoarthritis or rheumatism), osteoporosis, cancer or malignant tumour (including leukaemia or lymphoma, but excluding minor skin cancers), stomach or duodenal ulcer or peptic ulcer, Parkinson disease, cataracts, hip fracture or femoral fracture. Logistic regression was used for multivariate analyses, including the 95% confidence intervals. All analyses were conducted with the statistical software package SAS (version 8.2).

## Results

The prevalence of the dependent variable 'forgone care' differs by country. Looking first at forgone care because of costs 'or' unavailability, between 2.42% (The Netherlands) and 9.85% (Greece) of the respondents report to have experienced this in the last twelve months. In order to assure that the estimates are not based on too small numbers, the following country-specific analyses are restricted to those countries with at least 150 cases (i.e. France, Germany, Greece, Italy and Sweden; total n = 14,178). Focusing on these five countries, the distribution of the dependent and independent variables is shown in table 1. Calculating the percentage of forgone care that can be attributed to costs, some differences can be seen between the countries, ranging from 49% in Sweden to 89% in Germany. The age and sex distribution is rather similar in all five countries, and so is the prevalence of chronic disease. Larger differences can be seen for self assessed health, with poor health being much more prevalent in Italy (12.68%) and Germany (12.59%) than in Greece (8.07%).

**Table 1:** Distribution of the dependent and independent variables

	France	Germany	Greece	Italy	Sweden
Sample Size	3,052	2,941	2,680	2,508	2,997

Response Rate (%)	81.0	63.4	63,1	54.5	46.9
Forgone Care					
- reason 1^a ^or 2^b ^(n, %)	238 (7.80)	184 (6.26)	264 (9.85)	181 (7.22)	172 (5.74)
- reason 1^a ^(n, %)	194 (6.36)	163 (5.54)	172 (6.42)	127 (5.06)	84 (2.80)
- proportion^c^	0.82	0.89	0.65	0.70	0.49
Sex (%)					
- men	44.89	46.58	46.31	44.90	46.95
Age (%)					
- 50 – 58	36.70	31.38	36.38	29.82	31.56
- 59 – 67	24.71	34.21	25.93	34.73	31.87
- 68 – 104	38.60	34.41	37.69	35.45	36.57
Self assessed health (%) ^d^					
- good	61.80	55.48	63.09	49.56	64.14
- fair	28.57	31.93	28.84	37.76	26.00
- poor	9.63	12.59	8.07	12.68	9.86
Chronic disease (%)^e^					
- yes	56.35	47.28	49.22	56.28	48.21
Income (Euro) ^c^					
- 1^st ^(low) ^f^	11,753	12,311	5,647	7,283	19,657
- 2^nd^	18,808	20,695	8,425	12,255	29,054
- 3^rd^	29,750	32,301	12,600	18,324	39,093
- 4^th^	54,304	53,859	21,554	31,707	57,627
- 5^th ^(high)	>54,304	>53,859	>21,554	>31,707	>57,627
- 4^th^/1^st^	4.62	4.37	3.82	4.35	2.93
- Mean	39,657	37,216	13,985	22,044	41,517
- Median	23,704	25,942	10,021	15,267	33,686

a) forgone care because of costs

b) forgone care because care is unavailable

c) (percentage of reason 1)/(percentage of reason 1 or 2)

d) good: very good or good; bad: bad or very bad

e) at least one disease among a list of 12 doctor diagnosed diseases (see methods)

f) adjusted gross household income per year (quintiles 1 to 4: upper limits)

The quintiles of the adjusted per capita income are calculated for each country separately, and the results show, for example, that the upper limit of the lowest quintile is much lower in Greece (5,647 Euro) than in Sweden (19,657 Euro). Large differences between the five countries can also be seen for the quotient 'upper limit of 1st quintile/lower limit of 5th quintile', indicating different degrees of income inequality. It is much smaller in Sweden (2.93) than in France (4.62), for example. The mean and median values of the adjusted per capita income again point to the fact that the level of income is very different in these countries, being particularly low in Greece. It is also important to point out that there are very few missing values for the variables presented here. Taking all 5 countries together (total n = 14,178), there are no missing values for age, sex and the income variable (missing values for income have been replaced in the SHARE data set, based on a sophisticated algorithm [see ]). Also, there are 92 missing values for self assessed health, 82 for chronic disease, and 121 each for forgone care because of costs and forgone care because of unavailability.

**Figure 1:**
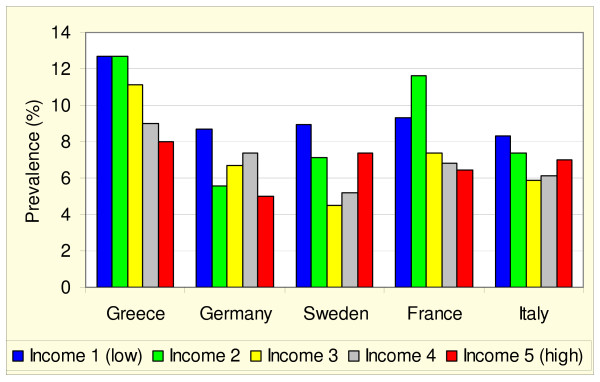
**Age and sex adjusted prevalence of forgone care because of costs or unavailability ^a^**. a) adjusted according to the age and sex distribution in the European Union.

In the next step, the prevalence of forgone care (because of costs 'or' unavailability) per income group is adjusted by age and sex (based on the age and sex distribution in the European Union). In all five countries, the prevalence of forgone care is higher in the lowest income group as compared with the highest income group (see figure 1). A regular stepwise pattern can only be seen for Greece, and a U-shape emerges for Sweden.

Controlling for age and sex in multivariate analysis, forgone care (because of costs 'or' unavailability) is still always higher in the lowest (as compared with the highest) income group (table 2). Some of these odds ratios are rather large. The most pronounced association can be seen for Greece, concerning the dose response pattern and the size of the odds ratios. The exception to the general pattern is Italy, showing no significant odds ratios for income. The odds ratio for the lowest income group is 1.34; the confidence interval (i.e. 0.86–2.09) clearly includes 1.0, though.

**Table 2:** Multivariate analysis (dependent variable: forgone care because of costs or unavailability)

	Odds Ratios (95% confidence interval)				
	France	Germany	Greece	Italy	Sweden
***Model 1 *^a^**					
Income-Quintiles					
- 5^th ^(high) (ref.)	1.00	1.00	1.00	1.00	1.00
- 4^th^	1.09 (0.73–1.62)	1.62 (1.03–2.54)	1.42 (0.95–2.12)	0.93 (0.61–1.43)	1.07 (0.70–1.64)
- 3^rd^	1.13 (0.74–1.71)	1.41 (0.87–2.28)	1.83 (1.22–2.74)	0.88 (0.54–1.42)	0.76 (0.44–1.34)
- 2^nd^	1.78 (1.18–2.69)	1.27 (0.74–2.18)	1.97 (1.29–3.02)	1.15 (0.70–1.89)	1.27 (0.78–2.09)
- 1^st ^(low)	1.55 (1.01–2.42)	2.18 (1.39–3.43)	2.18 (1.45–3.27)	1.34 (0.86–2.09)	1.81 (1.14–2.86)
***Model 2 *^b^**					
(n)^c^	(238)	(184)	(264)	(181)	(172)
Income-Quintiles					
- 5^th ^(high) (ref.)	1.00	1.00	1.00	1.00	1.00
- 4^th^	1.09 (0.73–1.63)	1.54 (0.98–2.43)	1.34 (0.89–2.01)	0.87 (0.57–1.35)	1.04 (0.68–1.59)
- 3^rd^	1.09 (0.71–1.66)	1.28 (0.79–2.08)	1.75 (1.16–2.63)	0.74 (0.45–1.21)	0.74 (0.42–1.29)
- 2^nd^	1.65 (1.09–2.51)	1.03 (0.60–1.78)	1.75 (1.14–2.70)	0.97 (0.59–1.61)	1.20 (0.73–1.97)
- 1^st ^(low)	1.37 (0.88–2.15)	1.74 (1.10–2.76)	2.00 (1.33–3.02)	1.07 (0.68–1.70)	1.58 (0.99–2.53)
SAH^d^					
- good (ref.)	1.00	1.00	1.00	1.00	1.00
- fair	1.60 (1.17–2.18)	2.45 (1.70–3.54)	2.43 (1.80–3.29)	2.88 (1.97–4.22)	1.68 (1.18–2.39)
- poor	2.03 (1.32–3.12)	5.00 (3.30–7.57)	4.18 (2.73–6.42)	5.78 (3.68–9.08)	1.96 (1.22–3.15)
Sex^e^					
- men (ref.)	1.00	1.00	1.00	1.00	1.00
- women	1.51 (1.14–2.00)	1.06 (0.78–1.44)	1.70 (1.28–2.25)	1.55 (1.12–2.16)	1.52 (1.09–2.11)

a) including: income, age, sex

b) including: income, age, sex, self assessed health

c) number of persons with forgone care (because of costs or unavailability)

d) reference group: good SAH

e) reference group: men

Additionally controlling for self assessed health (see model 2) reduces these odds ratios to some extent, but significant odds ratios remain. Concerning Sweden, forgone care in the lowest income group is 1.58 times as high as in the highest income group. For Germany, this odds ratio reaches 1.74, and for Greece 2.00. Again, a dose response pattern is seen mainly for Greece. Also, in four countries (i.e. France, Greece, Italy and Sweden) this risk is higher for women than for men. Large odds ratios can be seen for self assessed health, indicating that less than good health is associated with forgone care in a dose response way. Concerning age, only few odds ratios reach the level of statistical significance (not shown in the table).

Restricting the analysis to 'forgone care due to costs' reveals similar associations (not shown in the table). Controlling for age, sex and self assessed health, the odds ratios for income group 1 (low) and 2 are: for France 1.49 (95% CI: 0.90–2.46) and 1.95 (95% CI: 1.23–3.08); for Germany 1.81 (95% CI: 1.09–2.98) and 1.18 (95% CI: 0.66–2.12); for Greece 1.64 (95% CI: 1.00–2.70) and 1.82 (95% CI: 1.01–3.03); for Italy 2.09 (95% CI: 1.22–3.58) and 1.67 (95% CI: 0.92–3.03); for Sweden 4.20 (95% CI: 2.13–8.27) and 3.03 (95% CI: 1.49–6.13). Thus, most odds ratios increase (as compared to those presented in table 2), and for Italy and Sweden the association between income and forgone care becomes much more pronounced.

In further analyses we restricted the sample to participants with chronic disease (table 3). The sample size becomes much smaller, and some odds ratios change considerably. The pattern seen in table 2 is still apparent, though, indicating more forgone care in the low income groups in all countries except Italy. Again, a dose response association is seen for Greece, and again comparing the lowest with the highest income group yields the highest odds ratios for Greece (1.95) and for Germany (1.98). Concerning Sweden, an opposite association emerges for the 3rd income group.

**Table 3:** Multivariate analysis for participants with a chronic disease ^a ^(dependent variable: forgone care because of costs or unavailability)

	Odds Ratios ^b ^(95% confidence interval)				
	France	Germany	Greece	Italy	Sweden
(n)^c^	(160)	(118)	(167)	(141)	(88)
Income-Quintiles					
- 5^th ^(high)	1.00	1.00	1.00	1.00	1.00
- 4^th^	0.73 (0.42–1.29)	1.42 (0.77–2.60)	1.26 (0.73–2.18)	0.80 (0.49–1.33)	0.59 (0.30–1.17)
- 3^rd^	1.25 (0.74–2.09)	1.29 (0.70–2.40)	1.68 (0.98–2.88)	0.71 (0.41–1.22)	0.38 (0.15–0.93)
- 2^nd^	1.68 (1.01–2.79)	1.29 (0.66–2.50)	1.68 (0.96–2.93)	0.87 (0.49–1.54)	1.04 (0.54–2.00)
- 1^st ^(low)	1.46 (0.85–2.52)	1.98 (1.08–3.63)	1.95 (1.11–3.40)	0.85 (0.49–1.48)	1.33 (0.72–2.48)

a) at least one disease among a list of 12 doctor diagnosed diseases (see methods)

b) model includes the independent variables income, age, sex, self assessed health

c) number of persons with forgone care (because of costs or unavailability)

## Discussion

Concerning persons aged 50 years or older, the results indicate that low income groups report forgone care usually more often than high income groups. The association between income and forgone care can be seen in four of the five countries included in these analyses (i.e. France, Germany, Greece and Sweden), with Italy being the only exception. The association is most pronounced in Germany and Greece. The prevalence of forgone care increases with decreasing self assessed health (in all five countries), and it is more prevalent among women than among men (in all five countries except Germany).

There are important differences between these five countries, for example concerning income, the country specific overall level of forgone care, and also concerning the intra-country association between forgone care and income. Sweden is characterized by high average income and small income inequalities, for example, and Greece by low average income and high income inequalities. In future studies it should be assessed whether there is a causal link between these characteristics and the level of forgone care.

The SHARE-Study provides a very good basis for an international overview concerning the association between household income and forgone care. The data have been collected in different Western European countries by a standard protocol, they refer to a recent time period (i.e. 2004), and they are well accepted in the public health community [18-23]. Several potential problems have to be taken into account, though. The SHARE dataset includes information on 'gross total annual household income', including many different sources of income [16]. It would also be important to have information on *net *household income, as this could reflect the available financial resources more directly. The decision of the SHARE team to focus on *gross *income is justified by the fact that the link between gross and net household income strongly differs between countries. Interpreting the analyses presented here, it has to be kept in mind, though, that gross income primarily represents the social status and not the available financial resources.

It could be argued that reporting bias is an important issue, as the analyses are based on self report. Future studies should try to include more objective measures of health and health care access. Sample size is rather small for some countries, restricting the possibility to conduct separate analyses for each country. Some response rates are quite low, leaving ample room for response bias, and it is difficult to assess the potential effects of this. It can be assumed, for example, that the response rate is particularly low in the low income group and in the group experiencing forgone care, and that this bias could lead to an under-estimation of the association between income and forgone care. In our study it was not possible to assess these potential biases empirically. Future studies should include more information on non-responders. It would also be very important to increase the sample size per country, as more detailed analyses are necessary e.g. for better understanding the social and the health consequences of forgone care.

## Conclusion

The general recommendation is to reduce the number of persons who claim that they did not receive medical care which they believed they had needed. Maybe this claim is not based on an objective need for health care, but it could still have adverse health effects. Subjective statements concerning forgone care are important in their own right, as the discomfort expressed by this statement could be an independent stressor, and as patient satisfaction could be a strong predictor of compliance [26]. If the claim is 'justified' by an objective need for health care, it is even more obvious to demand that forgone care should be reduced.

The next step should be to define the population groups with a particularly high prevalence of forgone care, and to conclude that efforts aimed at reducing forgone care should focus on those groups most affected. Concerning sex and household income there are some clear country specific differences. They indicate the need for developing specific policy recommendations for each country, according to the specific health care system. For each country it would be important, for example, to assess the association between forgone care on one hand and waiting lists and referrals to specialist care on the other.

We need to know more about the specific reasons that lie behind an answer indicating forgone care. Otherwise it will hardly be possible to understand the inter-country differences, and to develop specific interventions aimed at reducing forgone care. Having more information on the potential effects of forgone care on health (and on health care costs) for different diseases would allow us to be much more specific about policy recommendations. Another important step would be to include a comparison across time, e.g. before and after an intervention that increases the financial barrier of health care utilization for the patient at the point of delivery. Such rarely published comparisons [27] could be very informative for policy makers. Last but not least, it would be important to study the association with out-of-pocket health care expenditures. It has repeatedly been shown for different countries that these expenditures are regressive, i.e. low income groups pay disproportionally more than high income groups [28-32]. Future studies should assess whether forgone care increases with increasing out-of-pocket health care expenditures, especially in low income groups.

The analyses presented here add empirical evidence to the inverse care law [14,15]. In a recent paper on "concepts and principles for tackling social inequities in health", M. Whitehead and G. Dahlgren [33] stress that access to health care could include three different problems: geographic access, economic access and cultural access. Indicators of geographic and cultural access could not be included here; the analyses focus on a key indicator for economic access (i.e. the independent variable 'income'). It can be assumed that geographic and cultural access is associated with economic access, but each topic has to be studied separately.

## Competing interests

The authors declare that they have no competing interests.

## Authors' contributions

AM has supervised the project and he has drafted and edited the manuscript. RK has carried out the statistical analyses and he has made substantial contributions to the manuscript. The contribution of OvdK was essential for understanding the SHARE dataset and for revising earlier drafts of the manuscript. AK and IS have made substantial contributions to the design and interpretation of the analyses. All authors read and approved the final manuscript.

## Pre-publication history

The pre-publication history for this paper can be accessed here:


